# Supplementary material for: Using large language models to suggest informative prior distributions in Bayesian regression analysis
Source: Sci Rep. 2025 Sep 29;15:33386. doi: 10.1038/s41598-025-18425-9 (PMC12480929; doi:10.1038/s41598-025-18425-9)
Supplement: Supplementary file 1 — Supplementary Information. [file 41598_2025_18425_MOESM1_ESM.pdf]

Supplementary Material for the Paper

Using Large Language Models to Suggest Informative Prior Distributions in Bayesian Regression Analysis

Michael A. Riegler[1], **Kristoffer Herland Hellton**[2], **Vajira Thambawita**[3], and **Hugo L. Hammer**[2,3,*]

[1]Simula Research Laboratory, Lysaker, Norway
[2]Department of Computer Science, OsloMet - Oslo Metropolitan University, Oslo, Norway
[3]Holistic Systems, Simula Metropolitan Center for Digital Engineering, Oslo, Norway
[*]Corresponding author, hugoh@oslomet.no

Introduction

In this supplementary material we present the complete prompts used in the examples in the paper. We also present the complete responses from the different LLMs for the two examples.

1 Heart Disease Example

1.1 Prompt

Listing 1 shows the complete prompt used in the heart disease example in the paper.

```
You are an expert in biostatistics and cardiovascular epidemiology. For the
logistic regression model provided below, which predicts coronary artery
disease (CAD), your task is to propose and justify suitable normally
distributed prior distributions for all regression parameters ($\beta_0,
\ldots, \beta_6$).

**Model Details:**
Response: $y \in \{0,1\}$ (0 = healthy, 1 = CAD)

Linear Predictor: $\eta = \beta_0 + \beta_1 \text{age} + \beta_2 \text{sex} +
\beta_3 \text{trestbps} + \beta_4 \text{chol} + \beta_5 \text{thalach} +
\beta_6 \text{oldpeak}$

Predictor Details:
* ‘age‘: in years * ‘sex‘: categorical (1 = male; 0 = female)
* ‘trestbps‘: resting blood pressure (in mm Hg on admission to the hospital)
* ‘chol‘: serum cholesterol in mg/dl
* ‘thalach‘: maximum heart rate achieved (beats per minute)
* ‘oldpeak‘: ST depression induced by exercise relative to rest (a numerical
value, unitless or mm)

**Your Response Should:**
1. **Leverage Knowledge & Simulate Tool Use**: Briefly state how you’ll use
your existing knowledge of CAD risk factors and logistic regression modelling
(simulating the consultation of relevant literature or databases for effect
sizes and typical parameter ranges) to inform your suggestions. Do not rely
your knowledge on the Cleveland Heart Disease data, but rely on other resources.
```

```
2. **Propose Multiple Prior Sets**: Generate at least two distinct sets of
prior distributions (e.g., "Suggestion A: Moderately Informative Priors based
on Domain Knowledge" and "Suggestion B: Weakly Informative / More Conservative
Priors").
3. **Detailed Justification for Each Parameter**: For each parameter ($\beta_0$
through $\beta_6$) within each suggested set, provide:
* The specific normal prior distribution: $N(\text{mean}, \text{standard
deviation}^2)$. Clearly state the mean and standard deviation.
* **Rationale for the Mean**: Explain the choice of the mean based on the
expected direction (positive/negative) and plausible magnitude of the
predictor's effect on the log-odds of CAD. Reference established
epidemiological knowledge or typical findings where appropriate. For $\beta_0$,
discuss its interpretation (log-odds for an individual with all predictor
values at zero) and how its prior mean is derived, considering this
interpretation.
* **Rationale for the Standard Deviation**: Justify the standard deviation,
linking it to the degree of uncertainty, the expected range of plausible effect
sizes (log-odds ratios), or common practices for weakly informative priors in
medical research.
4. **Comparative Evaluation & Weighting**:
* Critically evaluate and compare the different sets of priors you've proposed.
Discuss their potential strengths (e.g., incorporating valuable existing
information, potentially leading to more stable estimates with smaller
datasets) and weaknesses (e.g., risk of prior-data conflict if domain knowledge
is inaccurate, potential for overly influencing results).
* Assign a relative weighting or confidence score (e.g., Suggestion A: 60%,
Suggestion B: 40%) to your proposed sets, and clearly explain the reasoning
behind this weighting. This weighting should reflect your expert opinion on
which set offers a better balance of informativeness and appropriate caution
for a typical epidemiological analysis of this kind, or under what
circumstances one might be preferred over the other.
```

Listing 1: Prompt Used in the heart disease example.

1.2 Response from Claude

```
I'll draw upon established cardiovascular epidemiological literature and
logistic regression modeling practices to propose suitable Bayesian priors for
this CAD prediction model.

1. Knowledge Base and Approach
I'll leverage well-established epidemiological findings from major studies
(Framingham Heart Study, MONICA project, meta-analyses) regarding CAD risk
factors. For logistic regression parameters, I'll consider that \beta
coefficients represent log-odds ratios, with typical effect sizes from
cardiovascular literature informing prior means, while uncertainty levels and
model stability considerations will guide prior standard deviations.

2. Prior Distribution Proposals
Suggestion A: Moderately Informative Priors (Domain Knowledge-Based)
* For the intercept, $\beta_0$, the proposed prior is $N(-2.5, 1.0^2)$. The
mean of -2.5 represents the log-odds of CAD for a reference individual (female,
age=0, all predictors=0). While not directly meaningful, this corresponds to a
```

```
baseline probability of ~7%, which is a reasonable starting point for young
females. The standard deviation of 1.0 allows for substantial uncertainty
around this central value.
* For age, $\beta_1$, the prior is $N(0.06, 0.02^2)$. The mean is based on
epidemiological studies showing a consistent ~6% increase in CAD odds per year
of age (log-OR $\approx$ 0.06). The standard deviation of 0.02 captures some
uncertainty while maintaining a strong prior belief in this positive
association.
* For sex, $\beta_2$, we suggest a prior of $N(0.7, 0.3^2)$. The mean reflects
the well-established finding that males have an approximately 2-fold higher
risk of CAD (log-OR $\approx$ 0.7). The standard deviation of 0.3 allows for
moderate uncertainty around this known effect size.
* For resting blood pressure (trestbps), $\beta_3$, the prior is $N(0.02,
0.01^2)$. This is based on hypertension studies indicating a ~2% increase in
CAD odds per mmHg. The small standard deviation of 0.01 reflects relatively
precise knowledge of blood pressure's effect.
* For cholesterol (chol), $\beta_4$, a prior of $N(0.008, 0.005^2)$ is used.
This represents a small but consistent positive effect (~0.8% per mg/dl) found
in cholesterol literature. The standard deviation of 0.005 reflects uncertainty
in the precise magnitude of this effect.
* For maximum heart rate (thalach), $\beta_5$, the prior is $N(-0.02, 0.01^2)$.
A higher maximum heart rate is indicative of better fitness and is protective
against CAD, justifying the negative mean. The standard deviation of 0.01
reflects moderate confidence in this negative association.
* For ST depression (oldpeak), $\beta_6$, the prior is $N(0.5, 0.2^2)$. As ST
depression is a strong marker for ischemia, the mean of 0.5 corresponds to a
~65% increase in odds per unit. The standard deviation of 0.2 allows for
uncertainty while maintaining a strong prior belief in the risk.


Suggestion B: Weakly Informative Priors (Conservative Approach)
* For the intercept, $\beta_0$, a more conservative prior of $N(-1.5, 2.0^2)$
is proposed. The mean is less specific about the baseline risk, and the larger
standard deviation of 2.0 provides substantial uncertainty and flexibility for
the model.
* For age, $\beta_1$, the prior is $N(0.04, 0.05^2)$. This uses a more
conservative estimate for the age effect, and the wider standard deviation of
0.05 allows for a broad range of possibilities, including a minimal effect.
* For sex, $\beta_2$, we suggest a prior of $N(0.5, 0.6^2)$. This acknowledges
the male excess risk but with less certainty. The large standard deviation of
0.6 allows for substantial uncertainty, including the possibility of minimal
gender differences.
* For resting blood pressure (trestbps), $\beta_3$, the prior is $N(0.01,
0.02^2)$. This assumes a more conservative effect of blood pressure, and the
standard deviation of 0.02 allows for the possibility of both minimal and
strong effects.
* For cholesterol (chol), $\beta_4$, a prior of $N(0.005, 0.01^2)$ is used to
represent a small expected effect. The wide standard deviation of 0.01 provides
a large range of uncertainty.
* For maximum heart rate (thalach), $\beta_5$, the prior is $N(-0.01, 0.02^2)$.
This expects a mild protective effect, but the standard deviation of 0.02 is
wide enough to allow for uncertainty, including the possibility of a positive
effect.
```

```
* For ST depression (oldpeak), $\beta_6$, the prior is $N(0.3, 0.4^2)$. This
uses a conservative mean for the effect, and the wide standard deviation of 0.4
allows for a broad range of plausible effect sizes.

3. Comparative Evaluation and Weighting

Suggestion A (Moderately Informative): 65% Confidence
Strengths:
* Incorporates decades of cardiovascular epidemiological research
* Likely to produce more stable estimates with moderate sample sizes
* Reduces impact of outliers and implausible parameter combinations
* Aligns with established clinical understanding
Weaknesses:
* Risk of prior-data conflict if study population differs from literature base
* May constrain model flexibility in discovering novel associations
* Could bias results if domain knowledge assumptions are incorrect
Suggestion B (Weakly Informative): 35% Confidence
Strengths:
* Greater model flexibility and data-driven inference
* Reduced risk of inappropriate prior influence
* Better suited for exploratory analysis or novel populations
* More conservative approach when domain knowledge uncertainty exists
Weaknesses:
* May produce less stable estimates with smaller datasets
* Loses opportunity to incorporate valuable existing knowledge
* Higher risk of overfitting or implausible parameter estimates
* May require larger sample sizes for reliable inference

4. Recommendation Rationale
I weight Suggestion A more heavily (65%) because:
* Established Evidence Base: CAD risk factors are among the most studied in
epidemiology, providing robust prior information
* Clinical Relevance: The moderately informative priors align with clinical
understanding and improve interpretability
* Practical Benefits: In typical epidemiological studies (n=500-2000), these
priors will enhance stability without overly constraining inference
* Sensitivity Analysis: The proposed standard deviations are large enough that
strong contradictory data will override the priors
When to prefer Suggestion B:
* Very large datasets (n>5000) where data dominates priors regardless
* Populations significantly different from established literature (e.g.,
non-Western populations)
* Exploratory studies investigating novel risk factor relationships
* When regulatory or publication requirements favor conservative prior
assumptions

Both approaches should ideally be compared through sensitivity analysis to
assess prior influence on posterior inference.
```

Listing 2: Heart disease example. Response from Claude

1.3 Response from Gemini

```
Okay, as an expert in biostatistics and cardiovascular epidemiology, I can
certainly help you propose and justify suitable normally distributed prior
distributions for your logistic regression model predicting Coronary Artery
Disease (CAD).

## 1. Leveraging Knowledge & Simulating Tool Use

To inform these prior suggestions, I will draw upon my existing knowledge base
regarding:
* **Established CAD risk factors**: The direction and general magnitude of
effects for age, sex, blood pressure, cholesterol, exercise capacity (thalach),
and ECG changes (oldpeak) are well-documented in cardiovascular epidemiology
literature.
* **Logistic regression properties**: The interpretation of coefficients as
log-odds ratios (log-ORs) and how their scale relates to odds ratios.
* **Typical parameter ranges**: I'll simulate consulting epidemiological
studies (e.g., Framingham Heart Study, INTERHEART, and various meta-analyses)
to get a sense of plausible effect sizes (ORs) for these predictors. For
instance, age is consistently a positive risk factor, with ORs often in the
range of 1.05-1.10 per year increase. Male sex also consistently shows higher
risk.
* **Prior specification practices**: Common strategies for developing
moderately informative and weakly informative priors in medical research,
including how to choose means and standard deviations to reflect existing
knowledge or appropriate skepticism.

I will use this simulated knowledge to propose two sets of priors: one
moderately informative, leveraging established findings, and another more
conservative, weakly informative set.

---

## 2. & 3. Proposed Prior Sets & Detailed Justification

Here are two distinct sets of prior distributions. For each parameter
$\beta_i$, the prior is $N(\text{mean}, \text{standard deviation}^2)$. The
standard deviation (SD) is provided; INLA requires precision ($1/\text{SD}^2$).

### Suggestion A: Moderately Informative Priors based on Domain Knowledge

These priors are centered around effect sizes that are plausible based on
existing literature, with standard deviations that allow for some uncertainty
but still constrain the parameters within generally accepted ranges. The
intercept prior is set assuming an individual with all predictor values at
zero, which is often an extrapolation and typically corresponds to a very low
probability of CAD.

**Intercept ($\beta_0$)**:
* **Prior**: $N(\text{mean}=-8.0, \text{SD}=1.5)$ so $N(-8.0, 1.5^2)$.
* **Rationale for Mean (-8.0)**: $\beta_0$ is the log-odds of CAD for an
individual with age=0, sex=female (0), trestbps=0, chol=0, thalach=0, and
oldpeak=0. Such an individual is biologically implausible for some variables
(e.g., age=0, trestbps=0) but represents the mathematical reference point. The
```

```
probability of CAD for this profile would be extremely low. A log-odds of -8.0
corresponds to a probability of $e^{-8.0} / (1 + e^{-8.0}) \approx 0.00033$.
This reflects a very low baseline risk for this reference.
* **Rationale for Standard Deviation (1.5)**: This SD allows the log-odds to
vary, roughly between -11.0 and -5.0 (mean $\pm 2 \times$ SD), corresponding to
probabilities from approximately $0.000017$ to $0.0067$. This range reflects
some uncertainty but keeps the baseline probability very low for the reference
individual.

**Age ($\beta_1$)**:
* **Prior**: $N(\text{mean}=0.067, \text{SD}=0.021)$ so $N(0.067, 0.021^2)$.
* **Rationale for Mean (0.067)**: Corresponds to an OR of $e^{0.067} \approx
1.07$ per year of age. This suggests a 7% increase in odds of CAD per year
increase in age, a commonly observed magnitude.
* **Rationale for Standard Deviation (0.021)**: This implies that plausible
log-ORs are roughly in the range $0.067 \pm 2 \times 0.021 = [0.025, 0.109]$,
corresponding to ORs from $1.025$ to $1.115$. This covers a typical range of
reported effects for age.

**Sex ($\beta_2$, 1=male, 0=female)**:
* **Prior**: $N(\text{mean}=0.69, \text{SD}=0.173)$ so $N(0.69, 0.173^2)$.
* **Rationale for Mean (0.69)**: Corresponds to an OR of $e^{0.69} \approx 2.0$
for males compared to females. Males are known to be at higher risk, and an OR
of 2 is a common estimate.
* **Rationale for Standard Deviation (0.173)**: Log-OR range $0.69 \pm 2 \times
0.173 = [0.344, 1.036]$, so ORs from $1.41$ to $2.82$. This reflects a
reasonable uncertainty around the male vs. female risk.

**Trestbps ($\beta_3$, resting blood pressure in mm Hg)**:
* **Prior**: $N(\text{mean}=0.01, \text{SD}=0.005)$ so $N(0.01, 0.005^2)$.
* **Rationale for Mean (0.01)**: Corresponds to an OR of $e^{0.01} \approx
1.01$ per mm Hg increase. This means a 1% increase in odds per mm Hg, or a 10%
increase per 10 mm Hg (OR $\approx 1.105$), which is epidemiologically
plausible.
* **Rationale for Standard Deviation (0.005)**: Log-OR range $0.01 \pm 2 \times
0.005 = [0.00, 0.02]$, so ORs from $1.00$ to $1.02$ per mm Hg. This captures
the expectation of a modest, positive effect.

**Chol ($\beta_4$, serum cholesterol in mg/dl)**:
* **Prior**: $N(\text{mean}=0.005, \text{SD}=0.0025)$ so $N(0.005, 0.0025^2)$.
* **Rationale for Mean (0.005)**: Corresponds to an OR of $e^{0.005} \approx
1.005$ per mg/dl increase. This implies a 0.5% increase in odds per mg/dl, or
about a 5.1% increase per 10 mg/dl (OR $\approx 1.051$), consistent with
literature.
* **Rationale for Standard Deviation (0.0025)**: Log-OR range $0.005 \pm 2
\times 0.0025 = [0.000, 0.010]$, so ORs from $1.000$ to $1.010$ per mg/dl.
Reflects a small but positive expected effect.

**Thalach ($\beta_5$, maximum heart rate achieved)**:
* **Prior**: $N(\text{mean}=-0.02, \text{SD}=0.01)$ so $N(-0.02, 0.01^2)$.
* **Rationale for Mean (-0.02)**: Corresponds to an OR of $e^{-0.02} \approx
0.98$ per unit increase in max heart rate. Higher max heart rate achieved
during exercise is generally associated with better cardiovascular fitness and
lower CAD risk. An OR of 0.98 per beat indicates a 2% decrease in odds.
```

```
* **Rationale for Standard Deviation (0.01)**: Log-OR range $-0.02 \pm 2 \times
0.01 = [-0.04, 0.00]$, so ORs from $0.96$ to $1.00$. This allows for a
protective effect or no effect.

**Oldpeak ($\beta_6$, ST depression induced by exercise relative to rest)**:
* **Prior**: $N(\text{mean}=0.588, \text{SD}=0.163)$ so $N(0.588, 0.163^2)$.
* **Rationale for Mean (0.588)**: Corresponds to an OR of $e^{0.588} \approx
1.8$. ST depression is a known indicator of myocardial ischemia and CAD. An OR
of 1.8 per unit increase is a substantial and plausible effect.
* **Rationale for Standard Deviation (0.163)**: Log-OR range $0.588 \pm 2
\times 0.163 = [0.262, 0.914]$, so ORs from $1.30$ to $2.50$. This captures the
expectation of a moderate to strong positive association.

---

### Suggestion B: Weakly Informative / More Conservative Priors

These priors are generally centered at 0 (no effect) for slope parameters, with
larger standard deviations to let the data primarily drive the estimates. The
intercept prior still reflects a low probability for the reference individual
($X=0$) but with more uncertainty than Suggestion A.

**Intercept ($\beta_0$)**:
* **Prior**: $N(\text{mean}=-7.0, \text{SD}=3.0)$ so $N(-7.0, 3.0^2)$.
* **Rationale for Mean (-7.0)**: Similar to Suggestion A, this reflects an
extremely low probability of CAD for the reference individual (all predictors
at 0). Log-odds of -7.0 implies $P(CAD) \approx 0.0009$.
* **Rationale for Standard Deviation (3.0)**: A larger SD than in Suggestion A,
allowing for more uncertainty. Log-odds range $-7.0 \pm 2 \times 3.0 = [-13.0,
-1.0]$. This corresponds to probabilities from $2.2 \times 10^{-6}$ to $0.269$.
This range is wide but centered on a low baseline.

**Age ($\beta_1$)**:
* **Prior**: $N(\text{mean}=0.0, \text{SD}=0.1)$ so $N(0.0, 0.1^2)$.
* **Rationale for Mean (0.0)**: Assumes no effect a priori, letting the data
determine the direction and magnitude.
* **Rationale for Standard Deviation (0.1)**: Allows for a wide range of
effects. Log-ORs roughly between $-0.2$ and $0.2$ (95% interval for $\beta$ is
$\pm 1.96 \times 0.1 \approx \pm 0.196$). This covers ORs from $e^{-0.196}
\approx 0.82$ to $e^{0.196} \approx 1.22$ per year. This is quite broad for a
per-year effect and thus weakly informative.

**Sex ($\beta_2$, 1=male, 0=female)**:
* **Prior**: $N(\text{mean}=0.0, \text{SD}=1.0)$ so $N(0.0, 1.0^2)$.
* **Rationale for Mean (0.0)**: Assumes no difference a priori.
* **Rationale for Standard Deviation (1.0)**: Log-ORs roughly between -1.96 and
1.96. This corresponds to ORs from $e^{-1.96} \approx 0.14$ (females much
higher risk) to $e^{1.96} \approx 7.1$ (males much higher risk). This is a
wide, weakly informative range for a binary predictor.

**Trestbps ($\beta_3$, resting blood pressure in mm Hg)**:
* **Prior**: $N(\text{mean}=0.0, \text{SD}=0.05)$ so $N(0.0, 0.05^2)$.
* **Rationale for Mean (0.0)**: Assumes no effect a priori.
```

```
* **Rationale for Standard Deviation (0.05)**: Log-ORs roughly between $-0.098$
and $0.098$. ORs from $e^{-0.098} \approx 0.90$ to $e^{0.098} \approx 1.10$ per
mm Hg. This is a wide range for a 1-unit change in blood pressure, making it
weakly informative.

**Chol ($\beta_4$, serum cholesterol in mg/dl)**:
* **Prior**: $N(\text{mean}=0.0, \text{SD}=0.02)$ so $N(0.0, 0.02^2)$.
* **Rationale for Mean (0.0)**: Assumes no effect a priori.
* **Rationale for Standard Deviation (0.02)**: Log-ORs roughly between
$-0.0392$ and $0.0392$. ORs from $e^{-0.0392} \approx 0.96$ to $e^{0.0392}
\approx 1.04$ per mg/dl. This range for a 1-unit change is sufficiently broad
to be considered weakly informative.

**Thalach ($\beta_5$, maximum heart rate achieved)**:
* **Prior**: $N(\text{mean}=0.0, \text{SD}=0.05)$ so $N(0.0, 0.05^2)$.
* **Rationale for Mean (0.0)**: Assumes no effect a priori.
* **Rationale for Standard Deviation (0.05)**: Same reasoning as for
‘trestbps‘. Log-ORs roughly between $-0.098$ and $0.098$. ORs from $0.90$ to
$1.10$ per unit change in max heart rate.

**Oldpeak ($\beta_6$, ST depression induced by exercise relative to rest)**:
* **Prior**: $N(\text{mean}=0.0, \text{SD}=1.0)$ so $N(0.0, 1.0^2)$.
* **Rationale for Mean (0.0)**: Assumes no effect a priori.
* **Rationale for Standard Deviation (1.0)**: Same reasoning as for ‘sex‘.
Log-ORs roughly between -1.96 and 1.96, allowing ORs from $0.14$ to $7.1$. This
is wide enough to be weakly informative for a predictor that can have a strong
effect.

---

## 4. Comparative Evaluation & Weighting

**Suggestion A: Moderately Informative Priors**
* **Strengths**:
    * Incorporates valuable, existing domain knowledge, which can lead to more
plausible and stable parameter estimates, especially with smaller datasets or
when multicollinearity is present.
    * Can improve the precision (narrower credible intervals) of estimates if
the prior knowledge is accurate.
    * Reflects a common approach in Bayesian analysis where prior information
is explicitly used.
* **Weaknesses**:
    * If the domain knowledge is inaccurate or not generalizable to the
specific dataset, these priors can introduce bias and lead to prior-data
conflict.
    * Results might be perceived as less "objective" if the priors heavily
influence the posterior.
    * Requires careful justification and sourcing of the prior means and
standard deviations.

**Suggestion B: Weakly Informative / More Conservative Priors**
* **Strengths**:
    * Generally considered more "objective" as they allow the data to
predominantly drive the posterior estimates.
```

```
    * Lower risk of introducing strong bias from inaccurate prior beliefs.
    * Often easier to specify and justify, especially when domain knowledge is
uncertain or contentious.
    * Good for sensitivity analyses to see how much results depend on prior
choices.
* **Weaknesses**:
    * May lead to wider credible intervals and less stable estimates,
particularly with sparse data.
    * Truly "uninformative" priors are difficult to achieve, and "weakly
informative" choices can still subtly influence results, especially for the
intercept or with complex models.
    * May not take advantage of well-established knowledge, potentially leading
to less efficient use of information.

**Relative Weighting / Confidence Score**:

* **Suggestion A (Moderately Informative): 65%**
* **Suggestion B (Weakly Informative): 35%**

**Reasoning for Weighting**:
I assign a higher weighting to **Suggestion A (Moderately Informative Priors)**
because the predictors included in the model are well-established risk factors
for CAD, and their general effect directions and magnitudes are supported by a
large body of epidemiological research. Using moderately informative priors in
this context is a sound scientific approach that leverages existing knowledge,
potentially leading to more robust and clinically relevant findings, especially
if the dataset is not exceptionally large. The key is that the "information"
comes from a consensus in the field.

**Suggestion B (Weakly Informative Priors)** is valuable as a sensitivity
analysis or if there's significant doubt about the applicability of general
knowledge to the specific study population. It provides a good baseline to
understand what the current data alone suggests. However, in a field like
cardiovascular epidemiology where much is already known, ignoring this
information entirely (by using very diffuse priors) might not be the most
efficient or impactful approach.

The choice between them can also depend on the study's aim:
* For **confirmatory studies** where effects are expected, moderately
informative priors are often appropriate.
* For **exploratory studies** or when challenging existing beliefs, weakly
informative priors might be preferred.

Given the typical context of predicting CAD with these standard variables,
incorporating some existing knowledge (Suggestion A) is often beneficial,
provided it's done transparently and thoughtfully.
```

Listing 3: Heart disease example. Response from Gemini

1.4 Response from ChatGPT

```
Below I first outline how I have drawn on domain knowledge and "simulated"
consulting the epidemiological literature to anchor plausible effect-size
```

```
ranges. Then I present two distinct prior-set proposals (A: Moderately
Informative; B: Weakly Informative), giving for each parameter the Normal(mean,
sd^2), plus a rationale for both mean and sd. Finally, I compare the two sets
and assign relative weights, and show the exact R-INLA code you could plug in
for each suggestion.

Leveraging domain knowledge ("Simulated Literature Consultation")

I have reviewed large cohort studies (e.g., Framingham, MESA) and meta-analyses
of traditional CAD risk factors to get a sense of typical log-odds per unit
change:
age: roughly OR $\approx$ 1.04-1.06 per year, which corresponds to log-OR
$\approx$ 0.04-0.06.
sex: OR $\approx$ 1.8-2.5, which corresponds to log-OR $\approx$ 0.6-0.9 (male
vs. female).
trestbps: very small per mm Hg, OR $\approx$ 1.002-1.005, log-OR $\approx$
0.002-0.005.
chol: per mg change OR $\approx$ 1.001-1.003, log-OR $\approx$ 0.001-0.003.
thalach: protective, OR $\approx$ 0.98-0.99 per bpm, log-OR $\approx$ -0.01 to
-0.02.
oldpeak: per 1 mm ST-depression OR $\approx$ 1.3-1.6, log-OR $\approx$
0.26-0.47.
For the intercept (intercept), I considered the baseline log-odds when all
predictors equal zero. While age = 0 is unrealistic, a very low baseline CAD
risk in a reference profile corresponds to about p $\approx$ 0.05, which gives
logit(p) $\approx$ -2.94.

Suggestion A: Moderately Informative Priors
intercept: N(-2.5, $1^2$). Mean chosen as slightly shrunk toward zero from the
baseline log-odds of -2.94 for uncertainty. SD=1 allows baseline probability
from about 0.02 to 0.20.
age: N(0.05, $0.02^2$). Mean based on published effects of 0.04-0.06 per year.
SD=0.02 allows 95\% of values within +/-0.04 of the mean.
sex: N(0.7, $0.3^2$). Mean based on male vs. female log-OR of 0.6-0.9. SD=0.3
covers OR values from about 1.0 to 3.3 with high probability.
trestbps: N(0.002, $0.005^2$). Mean reflects per mm Hg effect of about
0.002-0.005 in log-odds. SD=0.005 gives a 95\% range of +/-0.01.
chol: N(0.002, $0.004^2$). Mean reflects per mg effect of about 0.001-0.003 in
log-odds. SD=0.004 covers +/-0.008.
thalach: N(-0.02, $0.01^2$). Mean based on protective effect of -0.01 to -0.02
per bpm. SD=0.01 covers -0.04 to 0.
oldpeak: N(0.4, $0.3^2$). Mean reflects OR $\approx$ 1.3-1.6 per unit ST
depression. SD=0.3 allows for a 95\% range from -0.2 to 1.0.

Suggestion B: Weakly Informative / More Conservative Priors
For all predictors except intercept: N(0, $2.5^2$), centered at zero to
represent no strong prior. SD=2.5 covers a very wide range of log-odds.
For intercept: N(0, $5^2$), centered at zero, with very large uncertainty,
covering baseline probabilities from <0.01 to >0.99.

Comparative Evaluation and Weighting
Suggestion A leverages published effect sizes to set tighter standard
deviations, leading to more stable estimates, especially in smaller datasets.
Risk of prior-data conflict is moderate, as true effects may differ in special
```

```
subgroups. This set is best when general CAD literature is trusted, for example
in middle-aged Western populations.
Suggestion B is very diffuse, letting the data speak more freely, and
minimizing the risk of prior-data conflict. However, this comes at the cost of
higher variance and potentially implausible estimates if the dataset is weak.
It is better suited when the sample is expected to differ strongly from the
populations studied in the literature.
Overall, I assign Suggestion A a weight of 60\% (preferred in typical
epidemiological analyses where established effect sizes are generalizable) and
Suggestion B 40\% (for extra-cautious or highly exploratory settings).
Suggestion A can be used by default, switching to Suggestion B if unexpected
effect directions are suspected or if the dataset is large enough for the data
to dominate the prior.
```

Listing 4: Heart disease example. Response from ChatGPT

2 Concrete Compressive Strength Example

2.1 Prompt

Listing 5 shows the complete prompt used in the concrete compressive strength example in the paper.

```
You are an expert in civil engineering. For the multiple linear regression
model provided below, which predicts concrete compressive strength (CCS), your
task is to propose and justify suitable normally distributed prior
distributions for all regression parameters ($\beta_0, \ldots, \beta_8$).

**Model Details:**
We consider a multivariate linear regression model: $CCS =  \beta_0 + \beta_1
Cement + \beta_2 Blast_Furnace_Slag + \beta_3 Fly_Ash + \beta_4 Water + \beta_5
Superplasticizer + \beta_6 Coarse_Aggregate + \beta_7 Fine_Aggregate + \beta_8
Age$

Response is CCS: Concrete compressive strength (MPa, megapascals): The
compressive strength of the concrete.

Explanatory variables:
Age (day) - How long the concrete has been allowed to cure before the
compressive strength test was performed. The time span for this variable is
from 1 day to 365 days.
All the variables below represent the amount included in the concrete:
Cement (component 1)(kg in a m^3 mixture)
Blast_Furnace_Slag (component 2)(kg in a m^3 mixture)
Fly_Ash (component 3)(kg in a m^3 mixture)
Water  (component 4)(kg in a m^3 mixture)
Superplasticizer (component 5)(kg in a m^3 mixture)
Coarse_Aggregate  (component 6)(kg in a m^3 mixture)
Fine_Aggregate (component 7)(kg in a m^3 mixture)

**Your Response Should:**
1. **Leverage Knowledge & Simulate Tool Use**: Briefly state how you'll use
your existing knowledge of CCS factors and multiple linear regression modelling
(simulating the consultation of relevant literature or databases for effect
sizes and typical parameter ranges) to inform your suggestions. Do not rely
your knowledge on the UCI Concrete Compressive Strength data, but rely on other
resources.
2. **Propose Multiple Prior Sets**: Generate at least two distinct sets of
prior distributions (e.g., "Suggestion A: Moderately Informative Priors based
on Domain Knowledge" and "Suggestion B: Weakly Informative / More Conservative
Priors").
3. **Detailed Justification for Each Parameter**: For each parameter ($\beta_0$
through $\beta_8$) within each suggested set, provide:
* The specific normal prior distribution: $N(\text{mean}, \text{standard
deviation}^2)$. Clearly state the mean and standard deviation.
* **Rationale for the Mean**: Explain the choice of the mean based on the
expected direction (positive/negative) and plausible magnitude of the
predictor's effect on CCS. Reference established civil engineering knowledge or
typical findings where appropriate. For $\beta_0$, discuss its interpretation
and how its prior mean is derived, considering this interpretation.
* **Rationale for the Standard Deviation**: Justify the standard deviation,
```

```
linking it to the degree of uncertainty, the expected range of plausible effect
sizes  or common practices for weakly informative priors in engineering
research.
4. **Comparative Evaluation & Weighting**:
* Critically evaluate and compare the different sets of priors you've proposed.
Discuss their potential strengths (e.g., incorporating valuable existing
information, potentially leading to more stable estimates with smaller
datasets) and weaknesses (e.g., risk of prior-data conflict if domain knowledge
is inaccurate, potential for overly influencing results).
* Assign a relative weighting or confidence score (e.g., Suggestion A: 60%,
Suggestion B: 40%) to your proposed sets, and clearly explain the reasoning
behind this weighting. This weighting should reflect your expert opinion on
which set offers a better balance of informativeness and appropriate caution
for a typical epidemiological analysis of this kind, or under what
circumstances one might be preferred over the other.
```

Listing 5: Prompt used in the concrete compressive strength example.

2.2 Response from Claude

Unfortunately we had not stored the complete response from Claude for this example. The response below is therefore from Claude Opus 4.1, while the computations used version 4.0. Some of the suggested paramters are therefore slightly different.

```
I'll propose and justify suitable normally distributed prior distributions for
your concrete compressive strength (CCS) regression model, drawing on civil
engineering principles and typical effect sizes from concrete materials
research.

1. Knowledge Base & Approach
I'll leverage established civil engineering knowledge about concrete mix
design, hydration chemistry, and strength development mechanisms. This
simulates consulting resources like ACI (American Concrete Institute)
standards, concrete technology handbooks, and meta-analyses of concrete
strength studies. Key considerations include:
Typical CCS values range from 10-80 MPa for standard concrete
Water-cement ratio is a critical negative predictor
Cementitious materials and age are primary positive predictors
Aggregate effects are generally moderate and positive

2. Proposed Prior Sets
Suggestion A: Moderately Informative Priors (Domain-Knowledge Based)
$\beta_0$ (Intercept): N(10, $10^2$)
Mean = 10 MPa: Represents a baseline strength when predictors are at reference
levels (though zero values aren't physically meaningful)
SD = 10: Allows substantial uncertainty around the baseline, accommodating
different concrete formulations
$\beta_1$ (Cement): N(0.08, $0.03^2$)
Mean = 0.08: Each kg/m$^3$ of cement increases strength by $\approx$ 0.08 MPa,
based on typical cement effectiveness
SD = 0.03: Moderate confidence, as cement's effect is well-established but
varies with quality
$\beta_2$ (Blast Furnace Slag): N(0.05, $0.04^2$)
```

```
Mean = 0.05: Supplementary cementitious material, $\approx$ 60\% as effective
as cement
SD = 0.04: Higher uncertainty due to variable reactivity and replacement ratios
$\beta_3$ (Fly Ash): N(0.03, $0.04^2$)
Mean = 0.03: Lower effectiveness than slag, contributes to long-term strength
SD = 0.04: Considerable uncertainty due to source variability
$\beta_4$ (Water): N(-0.15, $0.05^2$)
Mean = -0.15: Negative effect - excess water creates porosity and reduces
strength
SD = 0.05: Well-established relationship with moderate uncertainty
$\beta_5$ (Superplasticizer): N(1.5, $1.0^2$)
Mean = 1.5: Strong positive effect per kg/m$^3$ (used in small quantities)
SD = 1.0: High uncertainty as effectiveness varies greatly with type and dosage
$\beta_6$ (Coarse Aggregate): N(0.02, $0.02^2$)
Mean = 0.02: Moderate positive contribution to mechanical strength
SD = 0.02: Relatively certain, as aggregate effects are well-studied
$\beta_7$ (Fine Aggregate): N(0.015, $0.02^2$)
Mean = 0.015: Slightly less impact than coarse aggregate
SD = 0.02: Similar uncertainty to coarse aggregate
$\beta_8$ (Age): N(0.3, $0.1^2$)
Mean = 0.3: Strength gain per day (logarithmic relationship typically better,
but linear approximation)
SD = 0.1: Moderate confidence in age effect

Suggestion B: Weakly Informative Priors (Conservative)
$\beta_0$ (Intercept): N(20, $20^2$)
Mean = 20 MPa: Centered on typical low-strength concrete
SD = 20: Very broad, allowing data to dominate
$\beta_1$ (Cement): N(0.05, $0.1^2$)
Mean = 0.05: Positive but conservative estimate
SD = 0.1: Large uncertainty, weakly informative
$\beta_2$ (Blast Furnace Slag): N(0.03, $0.1^2$)
Mean = 0.03: Mildly positive expectation
SD = 0.1: High uncertainty
$\beta_3$ (Fly Ash): N(0.02, $0.1^2$)
Mean = 0.02: Small positive effect
SD = 0.1: High uncertainty
$\beta_4$ (Water): N(-0.1, $0.15^2$)
Mean = -0.1: Negative but less assertive
SD = 0.15: Very broad uncertainty
$\beta_5$ (Superplasticizer): N(1.0, $2.0^2$)
Mean = 1.0: Positive but conservative
SD = 2.0: Very high uncertainty
$\beta_6$ (Coarse Aggregate): N(0.01, $0.05^2$)
Mean = 0.01: Small positive effect
SD = 0.05: Broad uncertainty
$\beta_7$ (Fine Aggregate): N(0.01, $0.05^2$)
Mean = 0.01: Small positive effect
SD = 0.05: Broad uncertainty
$\beta_8$ (Age): N(0.2, $0.3^2$)
Mean = 0.2: Positive age effect
SD = 0.3: Very broad uncertainty

3. Comparative Evaluation & Weighting
```

```
Suggestion A Strengths:
* Incorporates valuable engineering knowledge
* Can improve estimation efficiency with smaller datasets
* Reflects established physical relationships
Suggestion A Weaknesses:
* Risk of prior-data conflict if local conditions differ
* May overly constrain estimates if domain assumptions are incorrect
Suggestion B Strengths:
* Allows data to dominate inference
* Reduces risk of prior misspecification
* More robust to unusual concrete formulations
Suggestion B Weaknesses:
* Less efficient with limited data
* May produce unstable estimates with small samples

Weighting: Suggestion A: 65\%, Suggestion B: 35\%
* I recommend Suggestion A for most applications because concrete science has
well-established relationships that should inform our analysis.
However, use Suggestion B if:
* Working with novel concrete formulations
* Sample size is large (n > 500)
* Prior sensitivity analysis is critical
```

Listing 6: Concrete compressive strength example. Response from Claude

2.3 Response from Gemini

```
As an expert in civil engineering, I will leverage my understanding of concrete
technology and the typical effects of its constituents on compressive strength,
combined with common practices in Bayesian regression modeling, to propose
suitable prior distributions. This involves simulating the consultation of
established literature and engineering handbooks for effect sizes and parameter
ranges, without referring to the specific UCI dataset mentioned.

---
## Proposed Prior Distributions for Concrete Compressive Strength Model
Parameters

Below are two distinct sets of normally distributed prior distributions for the
regression parameters ($\beta_0, \dots, \beta_8$) of the model:
$CCS = \beta_0 + \beta_1 \text{Cement} + \beta_2 \text{Blast_Furnace_Slag} +
\dots + \beta_8 \text{Age}$

### Suggestion A: Moderately Informative Priors based on Domain Knowledge
These priors are based on established civil engineering knowledge regarding the
expected direction and magnitude of influence of each component on Concrete
Compressive Strength (CCS).

For each parameter, the prior is specified as $N(\text{mean}, \text{standard
deviation}^2)$.

1.  $\beta_0$ (Intercept)
    * **Prior**: $N(5, 10^2)$ (Mean = 5 MPa, SD = 10 MPa)
```

```
    * **Rationale for Mean**: The intercept $\beta_0$ represents the predicted
CCS when all predictor quantities are zero. In reality, zero quantities of all
components would not form concrete. Thus, the intercept is largely an
extrapolation point or a baseline adjustment. A small positive value (5 MPa) is
chosen, imagining a hypothetical scenario or acknowledging that concrete
mixtures usually achieve some minimal strength very rapidly. If predictors were
centered, this prior would be centered around the mean CCS.
    * **Rationale for Standard Deviation**: An SD of 10 MPa provides a
plausible range (approx. 5 $\pm$ 20 MPa, i.e., -15 to 25 MPa in a 95\%
interval), accommodating some uncertainty in this extrapolated value and
allowing the data to adjust it considerably.

2.  $\beta_1$ (Cement)
    * **Prior**: $N(0.10, 0.05^2)$ (Mean = 0.10 MPa/(kg/m$^3$), SD = 0.05
MPa/(kg/m$^3$))
    * **Rationale for Mean**: Cement is the primary binder; increasing cement
content generally **increases** CCS. An increase of 1 kg/m$^3$ of cement is
expected to increase CCS by approximately 0.10 MPa, based on typical mix design
sensitivities where an additional 50-100 kg/m$^3$ of cement can increase 28-day
strength by 5-10 MPa.
    * **Rationale for Standard Deviation**: An SD of 0.05 allows the effect to
plausibly range from 0 to 0.20 MPa/(kg/m$^3$), reflecting that the exact
sensitivity varies with the overall mix design.

3.  $\beta_2$ (Blast Furnace Slag - BFS)
    * **Prior**: $N(0.06, 0.04^2)$ (Mean = 0.06 MPa/(kg/m$^3$), SD = 0.04
MPa/(kg/m$^3$))
    * **Rationale for Mean**: BFS is a supplementary cementitious material
(SCM) that typically enhances long-term strength. Its contribution per kg might
be slightly less than Portland cement, especially at earlier ages. A positive
effect of 0.06 MPa/(kg/m$^3$) is proposed.
    * **Rationale for Standard Deviation**: An SD of 0.04 allows the effect to
range (approx. -0.02 to 0.14 MPa/(kg/m$^3$)), acknowledging that BFS can
sometimes slightly reduce very early strength or have a more pronounced
positive effect depending on activation and curing.

4.  $\beta_3$ (Fly Ash - FA)
    * **Prior**: $N(0.05, 0.04^2)$ (Mean = 0.05 MPa/(kg/m$^3$), SD = 0.04
MPa/(kg/m$^3$))
    * **Rationale for Mean**: Fly Ash, another SCM, also generally contributes
positively to CCS, particularly at later ages, through pozzolanic reactions.
Its effect is often comparable to or slightly less than BFS.
    * **Rationale for Standard Deviation**: Similar to BFS, an SD of 0.04
(allowing approx. -0.03 to 0.13 MPa/(kg/m$^3$)) reflects variability in its
effectiveness based on ash quality and mix proportions.

5.  $\beta_4$ (Water)
    * **Prior**: $N(-0.15, 0.07^2)$ (Mean = -0.15 MPa/(kg/m$^3$), SD = 0.07
MPa/(kg/m$^3$))
    * **Rationale for Mean**: Water is essential for hydration, but excess
water (increasing the water-cement ratio) **decreases** CCS. A 1 kg/m$^3$
increase in water, holding other components constant, is expected to decrease
strength.
```

```
    * **Rationale for Standard Deviation**: An SD of 0.07 allows the effect to
range (approx. -0.29 to -0.01 MPa/(kg/m$^3$)), capturing typical sensitivities
related to w/c ratio changes.

6.  $\beta_5$ (Superplasticizer - SP)
    * **Prior**: $N(0.25, 0.15^2)$ (Mean = 0.25 MPa/(kg/m$^3$), SD = 0.15
MPa/(kg/m$^3$))
    * **Rationale for Mean**: Superplasticizers are used in small quantities.
They improve workability, enabling water reduction (which indirectly increases
strength) or enhancing particle dispersion for more efficient hydration. A
positive direct effect per kg is expected.
    * **Rationale for Standard Deviation**: An SD of 0.15 (allowing approx.
-0.05 to 0.55 MPa/(kg/m$^3$)) reflects that the effect can vary; sometimes it's
minimal if not linked to water reduction, sometimes more substantial.

7.  $\beta_6$ (Coarse Aggregate - CA)
    * **Prior**: $N(0.02, 0.02^2)$ (Mean = 0.02 MPa/(kg/m$^3$), SD = 0.02
MPa/(kg/m$^3$))
    * **Rationale for Mean**: Coarse aggregates form the structural skeleton.
While their quality matters significantly, their per-unit-mass effect in a
linear model might be small but generally positive, assuming good quality
aggregate.
    * **Rationale for Standard Deviation**: An SD of 0.02 (allowing approx.
-0.02 to 0.06 MPa/(kg/m$^3$)) reflects that the impact of quantity can be minor
or even slightly negative if it compromises workability or paste content
excessively.

8.  $\beta_7$ (Fine Aggregate - FA)
    * **Prior**: $N(0.02, 0.02^2)$ (Mean = 0.02 MPa/(kg/m$^3$), SD = 0.02
MPa/(kg/m$^3$))
    * **Rationale for Mean**: Fine aggregates are crucial for workability and
filling voids. Similar to coarse aggregates, a small positive per-unit-mass
effect is anticipated.
    * **Rationale for Standard Deviation**: An SD of 0.02 provides a similar
plausible range as for coarse aggregate.

9.  $\beta_8$ (Age)
    * **Prior**: $N(0.30, 0.15^2)$ (Mean = 0.30 MPa/day, SD = 0.15 MPa/day)
    * **Rationale for Mean**: CCS **increases** with age due to ongoing
hydration. The rate of gain is higher initially. A linear coefficient over
1-365 days represents an average daily increase. An average of 0.30 MPa/day is
a plausible value reflecting overall strength development.
    * **Rationale for Standard Deviation**: An SD of 0.15 (allowing approx. 0
to 0.6 MPa/day) acknowledges that the linear rate can vary significantly
depending on the mix design and the specific time window most represented in
the data.

---
### Suggestion B: Weakly Informative / More Conservative Priors
These priors are more conservative, generally centered at zero for slope
parameters (indicating no effect a priori) with larger standard deviations.
This allows the data to play a more dominant role in determining the parameter
estimates.
```

```
1.  $\beta_0$ (Intercept)
    * **Prior**: $N(20, 50^2)$ (Mean = 20 MPa, SD = 50 MPa)
    * **Rationale for Mean**: Instead of zero, the mean is set to a low but
plausible average CCS value (e.g., 20 MPa), recognizing that CCS is inherently
positive.
    * **Rationale for Standard Deviation**: A very large SD of 50 MPa makes
this prior diffuse over a wide range of CCS values (approx. 20 $\pm$ 100 MPa),
imposing minimal prior belief on the intercept's specific value.

2.  $\beta_1$ (Cement)
    * **Prior**: $N(0, 0.2^2)$ (Mean = 0 MPa/(kg/m$^3$), SD = 0.2
MPa/(kg/m$^3$))
    * **Rationale for Mean**: Assumes no effect a priori, letting the data
drive the estimate.
    * **Rationale for Standard Deviation**: An SD of 0.2 allows for a wide
range of effects (approx. $\pm$0.4 MPa/(kg/m$^3$)), covering plausible positive
values and even small negative ones if the data suggests it (though unlikely
for cement).

3.  $\beta_2$ (Blast Furnace Slag - BFS)
    * **Prior**: $N(0, 0.2^2)$ (Mean = 0 MPa/(kg/m$^3$), SD = 0.2
MPa/(kg/m$^3$))
    * **Rationale for Mean**: Assumes no effect a priori.
    * **Rationale for Standard Deviation**: An SD of 0.2 provides a wide
plausible range (approx. $\pm$0.4 MPa/(kg/m$^3$)).

4.  $\beta_3$ (Fly Ash - FA)
    * **Prior**: $N(0, 0.2^2)$ (Mean = 0 MPa/(kg/m$^3$), SD = 0.2
MPa/(kg/m$^3$))
    * **Rationale for Mean**: Assumes no effect a priori.
    * **Rationale for Standard Deviation**: An SD of 0.2 provides a wide
plausible range (approx. $\pm$0.4 MPa/(kg/m$^3$)).

5.  $\beta_4$ (Water)
    * **Prior**: $N(0, 0.2^2)$ (Mean = 0 MPa/(kg/m$^3$), SD = 0.2
MPa/(kg/m$^3$))
    * **Rationale for Mean**: Assumes no effect a priori.
    * **Rationale for Standard Deviation**: An SD of 0.2 allows for a
substantial negative effect (as expected) but also positive ones (approx.
$\pm$0.4 MPa/(kg/m$^3$)).

6.  $\beta_5$ (Superplasticizer - SP)
    * **Prior**: $N(0, 0.5^2)$ (Mean = 0 MPa/(kg/m$^3$), SD = 0.5
MPa/(kg/m$^3$))
    * **Rationale for Mean**: Assumes no effect a priori.
    * **Rationale for Standard Deviation**: SP is used in smaller quantities,
so its per-kg effect could be larger. An SD of 0.5 (approx. $\pm$1.0
MPa/(kg/m$^3$)) gives a broad range.

7.  $\beta_6$ (Coarse Aggregate - CA)
    * **Prior**: $N(0, 0.1^2)$ (Mean = 0 MPa/(kg/m$^3$), SD = 0.1
MPa/(kg/m$^3$))
    * **Rationale for Mean**: Assumes no effect a priori.
```

```
    * **Rationale for Standard Deviation**: The effect per kg is expected to be
smaller than that of cementitious materials or water. SD of 0.1 (approx.
$\pm$0.2 MPa/(kg/m$^3$)) provides a reasonable range.

8.  $\beta_7$ (Fine Aggregate - FA)
    * **Prior**: $N(0, 0.1^2)$ (Mean = 0 MPa/(kg/m$^3$), SD = 0.1
MPa/(kg/m$^3$))
    * **Rationale for Mean**: Assumes no effect a priori.
    * **Rationale for Standard Deviation**: Similar to coarse aggregate, an SD
of 0.1 allows for a modest range of effects.

9.  $\beta_8$ (Age)
    * **Prior**: $N(0, 1^2)$ (Mean = 0 MPa/day, SD = 1 MPa/day)
    * **Rationale for Mean**: Assumes no effect a priori.
    * **Rationale for Standard Deviation**: An SD of 1 MPa/day (approx. $\pm$2
MPa/day) allows for a wide range of positive (expected) or even negative linear
trends in strength with age, making it quite uninformative about the direction.

---
## Comparative Evaluation & Weighting

**Suggestion A: Moderately Informative Priors**
* **Strengths**:
    * Incorporates valuable existing domain knowledge from civil engineering,
potentially leading to more stable and realistic estimates, especially with
smaller datasets.
    * Priors are centered on expected effect directions (e.g., positive for
cement, negative for water), which can guide the model appropriately.
    * May result in faster convergence of MCMC samplers in Bayesian estimation.
* **Weaknesses**:
    * Risk of prior-data conflict if the domain knowledge embedded in the
priors is inaccurate for the specific dataset being analyzed.
    * Could unduly influence results if the priors are too narrow
(overconfident) and the data suggests otherwise.

**Suggestion B: Weakly Informative / More Conservative Priors**
* **Strengths**:
    * Reduces the risk of imposing incorrect prior beliefs on the model.
    * "Lets the data speak for itself" more, which is often preferred when
substantial data is available or when exploring new relationships.
    * Generally considered more objective or robust if prior knowledge is
uncertain.
* **Weaknesses**:
    * May require larger datasets to achieve stable and precise estimates
compared to informative priors.
    * Can sometimes lead to estimates in regions of the parameter space that
are physically implausible if the data is noisy or sparse, especially for
priors centered at zero for effects known to be strong and unidirectional.
    * The $N(0, \sigma^2)$ form for effects known to be strongly positive
(e.g., Age, Cement) or negative (e.g., Water) might seem counterintuitive, but
it is a common way to express weak information about the magnitude while being
open to directions (though the data will likely confirm the expected direction).

**Relative Weighting/Confidence Score:**
```

```
* **Suggestion A (Moderately Informative): 65\%**
* **Suggestion B (Weakly Informative): 35\%**

**Reasoning for Weighting:**

In civil engineering, particularly concerning well-understood materials like
concrete, there's a substantial body of established knowledge. The factors
influencing concrete strength are generally known. Therefore, **Suggestion A**,
which carefully incorporates this domain knowledge, is likely to provide a
better starting point for many analyses, potentially leading to more robust and
interpretable models, especially if the dataset isn't massive. The chosen means
reflect expected behavior, and the standard deviations are set to be reasonably
inclusive of variations. This approach aligns well with the Bayesian philosophy
of combining prior knowledge with data.

However, **Suggestion B** remains valuable. It is preferred when there's a
desire to be maximally data-driven, when the specific concrete mixes under
study might behave unusually, or when rigorously testing existing knowledge
against new data without strong presuppositions. The wider standard deviations
and zero-centering for slopes in Suggestion B ensure that the posterior
estimates will be heavily influenced by the likelihood.

**Preference Context:**
* **Choose Suggestion A if**: You have a reasonably sized dataset and trust
established civil engineering principles for concrete. It can help in
regularizing the model and yielding more precise estimates.
* **Choose Suggestion B if**: You have a very large dataset, or if you suspect
the concrete mixes in your data might have unique characteristics not
well-captured by general knowledge, or if you aim for results less influenced
by prior assertions.

Ultimately, sensitivity analysis using different prior specifications is often
a good practice in Bayesian modeling to understand the influence of prior
choices on the final conclusions.
```

Listing 7: Concrete compressive strength example. Response from Gemini

2.4 Response from ChatGPT

```
Below I first outline how I have drawn on civil-engineering domain knowledge
(simulating a brief literature/database check) to inform plausible effect sizes
for each predictor. I then give two distinct sets of normally distributed priors
Suggestion A (moderately informative, reflecting typical observed effects)
and Suggestion B (weakly informative, conservative) with full justifications
for each beta. Finally, I compare the two and assign relative weights.

1. Domain Knowledge "Check" (simulated)
Concrete strength typically increases with cement, slag, fly-ash (pozzolanic
materials),
superplasticizer and age, and decreases with water content (w/c ratio effect)
and,
to a small extent, with certain aggregates.
```

```
Published studies often report a per-kg effect of cement around 0.02-0.05
MPa/kg,
water around -0.08 to -0.12 MPa/kg, superplasticizer around 0.3-0.7 MPa/kg,
and age around 0.02-0.05 MPa/day (early plateauing notwithstanding).
Effects of aggregates are usually very small (fractions of a MPa per kg)
and can plausibly be centered near zero with tighter uncertainty.

2. Suggestion A: Moderately Informative Priors
Intercept: N(20, 10^2). With all ingredients at zero (unrealistic) but
centering roughly
around a mid-range CCS (~20 MPa) seen in practice. Sigma = 10 MPa allows
intercept to vary
from very low to very high strengths.
Cement: N(0.03, 0.02^2). 0.03 MPa per kg is in the middle of reported 0.02-0.05
MPa/kg effects.
Blast_Furnace_Slag: N(0.02, 0.02^2). Slightly lower reactivity than cement.
Fly_Ash: N(0.01, 0.015^2). Smaller pozzolanic effect.
Water: N(-0.10, 0.05^2). Each extra kg lowers strength by ~0.10 MPa.
Superplasticizer: N(0.50, 0.30^2). Improves flow and packing, with high
uncertainty.
Coarse_Aggregate: N(0.002, 0.002^2). Very small positive effect.
Fine_Aggregate: N(-0.001, 0.002^2). Slight negative or neutral effect.
Age: N(0.03, 0.01^2). Early strength gain around 0.03 MPa/day.

3. Suggestion B: Weakly Informative (Conservative)
Intercept: N(0, 100^2). Very broad prior: allows intercept anywhere from -200
to +200 MPa.
All slopes: N(0, 1^2). Reflects prior ignorance: each kg or day could shift CCS
by
up to roughly +/- 2 MPa.

4. Comparative Evaluation and Weighting
Suggestion A is moderately informative; it reflects real-world effect sizes and
can produce more stable posteriors and faster convergence. It carries moderate
risk of prior-data conflict if domain knowledge is wrong.
Suggestion B is very weak; it has minimal influence on the likelihood and low
risk of prior-data conflict. It is best for exploratory modeling or when prior
knowledge is highly uncertain.

Expert weighting: Suggestion A = 70%, Suggestion B = 30%.
Choose the set that best matches your modeling context.
```

Listing 8: Concrete compressive strength example. Response from ChatGPT
